# Migration and Differentiation of Neural Stem Cells Diverted From the Subventricular Zone by an Injectable Self-Assembling β-Peptide Hydrogel

**DOI:** 10.3389/fbioe.2019.00315

**Published:** 2019-11-08

**Authors:** Sepideh Motamed, Mark P. Del Borgo, Kun Zhou, Ketav Kulkarni, Peter J. Crack, Tobias D. Merson, Marie-Isabel Aguilar, David I. Finkelstein, John S. Forsythe

**Affiliations:** ^1^Department of Materials Science and Engineering, Monash Institute of Medical Engineering, Monash University, Clayton, VIC, Australia; ^2^Department of Biochemistry and Molecular Biology, Biomedicine Discovery Institute, Monash University, Clayton, VIC, Australia; ^3^Department of Pharmacology, The University of Melbourne, Parkville, VIC, Australia; ^4^Australian Regenerative Medicine Institute, Monash University, Clayton, VIC, Australia; ^5^Florey Institute of Neuroscience and Mental Health, The University of Melbourne, Parkville, VIC, Australia

**Keywords:** brain repair, neural stem cells, peptide hydrogels, self-assembly, neural tissue engineering

## Abstract

Neural stem cells, which are confined in localised niches are unable to repair large brain lesions because of an inability to migrate long distances and engraft. To overcome these problems, previous research has demonstrated the use of biomaterial implants to redirect increased numbers of endogenous neural stem cell populations. However, the fate of the diverted neural stem cells and their progeny remains unknown. Here we show that neural stem cells originating from the subventricular zone can migrate to the cortex with the aid of a long-lasting injectable hydrogel within a mouse brain. Specifically, large numbers of neuroblasts were diverted to the cortex through a self-assembling β-peptide hydrogel that acted as a tract from the subventricular zone to the cortex of transgenic mice (NestinCreER^T2^:R26eYFP) in which neuroblasts and their progeny are permanently fluorescently labelled. Moreover, neuroblasts differentiated into neurons and astrocytes 35 days post implantation, and the neuroblast-derived neurons were Syn1 positive suggesting integration into existing neural circuitry. In addition, astrocytes co-localised with neuroblasts along the hydrogel tract, suggesting that they assisted migration and simulated pathways similar to the native rostral migratory stream. Lower levels of astrocytes were found at the boundary of hydrogels with encapsulated brain-derived neurotrophic factor, comparing with hydrogel implants alone.

## Introduction

Brain lesions are a consequence of physical injury, stroke and neurodegeneration (Lindvall et al., 2004; Hyder et al., 2007; Eltzschig and Eckle, 2011; Hernández-Ortega et al., 2011) resulting in severe neurological disabilities (Orive et al., 2009). Current treatments are associated with preserving healthy neural tissue and there are no clinical treatments that promote regeneration and fully restore lost function (Pettikiriarachchi et al., 2010). While cell transplantation is an important strategy to replace lost neural tissue, issues with immune rejection, poor engraftment, ethical issues of embryonic cell sources and teratoma formation must first be resolved (Kondziolka et al., 2000; Master et al., 2007; Li et al., 2008; Hwang et al., 2010; Yasuda et al., 2010; Wang et al., 2012; Kang et al., 2014). Harnessing the regenerative power of the brain by using endogenous cells is therefore highly attractive.

Neural progenitor cells are continuously being produced in the adult brain, but their genesis is confined to the subgranular zone and the subventricular zone (SVZ) (Ma et al., 2009). Following brain injury, neural progenitor cells migrate into the injured region where they attempt differentiation and repair (Rennert et al., 2012). However, the brain's intrinsic repair mechanisms are largely ineffective, especially in the case of large lesions. The implications for the patient are therefore serious, manifesting in drastic and permanent disabilities.

In the healthy adult brain, neural stem cells (NSCs) residing in the SVZ divide and transit into amplifying cells which consequently differentiate into neuroblasts. The neuroblasts slide as neuronal chains along the rostral migratory stream (RMS) toward the olfactory bulb, where they differentiate into neurons and integrate in the granule and periglomerular layers into neural networks (Doetsch et al., 1999; Alvarez-Buylla and Lim, 2004; Ghashghaei et al., 2007; Whitman and Greer, 2009). Directed neuroblast migration through the RMS proceeds without dispersing into the surrounding tissue, however, this is a complicated process requiring a combination of cellular structures, signals, and cues (Lalli, 2014). Neuroblast migration from the SVZ is mediated by insulin-like growth factor I and fibroblast growth factor 2 (Hurtado-Chong et al., 2009). In addition, the chain of migrating neuroblasts use blood vessels, which are located in high density and aligned along the RMS, as a physical support to move forward (Bovetti et al., 2007). Vascular endothelial growth factor (VEGF), secreted by astrocytes surrounding the RMS, induces blood vessel generation and therefore indirectly regulates neuroblast motility (Bozoyan et al., 2012). Inhibition of brain-derived neurotrophic factor (BDNF) causes disruption of neuroblast migration throughout the RMS (Zhou et al., 2015). BDNF, secreted by blood vessels (Snapyan et al., 2009), promotes neuroblast movement via the p75^NTR^ receptor and increases the number of migratory cells (Chiaramello et al., 2007). It has also been shown that BDNF increases the displacement distance of neuroblasts by promoting neuroblasts to switch from a mitotic phase to a motile phase (Snapyan et al., 2009). Migrating neuroblasts are isolated from the surrounding tissue via glial tubes made by astrocytes, preventing the dispersion of cells from the stream and guiding them in the direction of the RMS (Ghashghaei et al., 2007).

In response to injury, the brain initiates a glial response (Fitch and Silver, 2008) and subsequently, the SVZ proliferates new neuroblasts, some of which re-direct from the SVZ toward the injured area to replace lost neurons and glia (Kernie and Parent, 2010; Saha et al., 2012), using signals such as stromal-cell-derived factor-1α (SDF1α) and metalloproteinases (MMP9) released from the local neurons and glia at the site of injury (Miller et al., 2005; Ghashghaei et al., 2007). Infusion of epidermal growth factor (EGF) and fibroblast growth factor 2 in a Parkinson's disease animal model elevated neural stem cell proliferation in the SVZ and enhancement of dopaminergic neurogenesis in the olfactory bulb (Winner et al., 2008). Neuroblasts migrating toward ischemia utilize a similar mechanism as used in the RMS to migrate, using blood vessels as physical guidance. Neuroblasts migrating toward ischemia have longer stationary phases in comparison to cells migrating through the RMS (Grade et al., 2013), which could be attributed to the low levels of endogenous BDNF. Low levels of endogenous BDNF after spinal cord injury is one of the important reasons for the hindrance of regeneration (Song et al., 2008). Therefore, by injecting exogenous BDNF into mouse injury models, neuroblast displacement toward ischemia doubled per hour by reducing the cell stationary phase periods (Grade et al., 2013).

We have previously investigated the feasibility of using scaffolds to promote neuroblast migration, which include the use of injectable gelatin-based hydrogels consisting of glial cell line-derived neurotrophic factor, electrospun poly-ε-caprolactone nanofibers releasing a BDNF mimetic, and graphene coated electrospun poly-ε-caprolactone fibres from the SVZ (Fon et al., 2014a,b; Zhou et al., 2016). The scaffolds were implanted into the brain in a way to impinge on the SVZ, and promoted neuroblast migration in all studies in comparison to injury only controls. Other studies have also demonstrated the possibility to redirect neuroblasts from RMS and SVZ by implanted scaffolds with specific signal cues such as β1 integrin, N-cadherin, VEGF, and nerve growth factor (Clark et al., 2016; Fujioka et al., 2017; Jinnou et al., 2018) being incorporated. However, to develop viable therapies to treat brain injuries, it is important to develop new injectable scaffolds that dramatically increase: (1) the number of migrating neuroblasts, (2) the migration distance, and (3) the persistency of migration over time. It is also important to determine the fate of the diverted neuroblasts, which the previous studies have not fully addressed.

**Figure 1:**
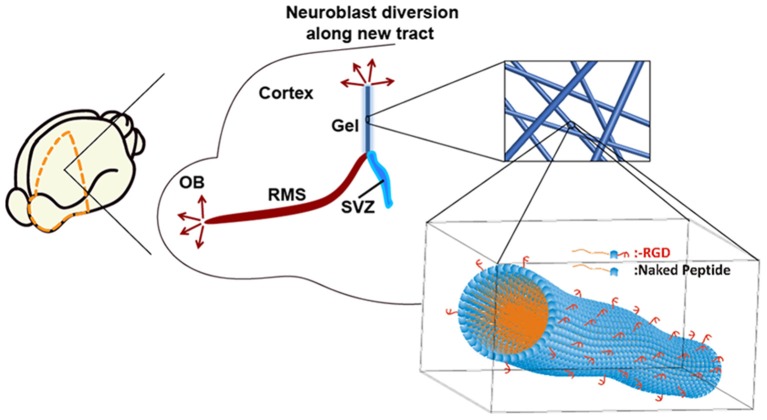
Neuroblasts originating from the SVZ migrate along the rostral migratory stream (RMS) to the olfactory bulb (OB). An implantable matrix composed of self-assembling β-peptide hydrogel forms a matrix tract between the SVZ and the cortex allowing diversion of neuroblast migration. Inset: Schematic of supramolecular self-assembly of N-acetylated β3-peptide functionalised with the integrin binding RGD epitope.

Previously, we introduced a new self-assembling peptide hydrogel composed exclusively of β-amino acids and a C_14_ hydrophobic acyl tail (C_14_-peptide hydrogel). The peptide self-assembled to form a stable and long-lasting hydrogel which was biocompatible with neuronal cells (Motamed et al., 2016). A dual-functionalized peptide hydrogel with an integrin binding arginylglycylaspartic acid (RGD) was also used to enhance cell attachment (RGD-peptide hydrogel). By mixing the C_14_- and RGD-peptides, the matrix was optimised to achieve high cell attachment *in vitro* (Kulkarni et al., 2016). In the present study, C_14_- and RGD-peptide (Kulkarni et al., 2016; Motamed et al., 2016) was used to encapsulate BDNF, and was implanted into the SVZ of tamoxifen inducible Nes-CreER^T2^: R26eYFP transgenic mice. NSCs residing in the SVZ of Nes-CreER^T2^:R26eYFP transgenic mice are permanently labelled when administered with tamoxifen, enabling tracking of these cells, throughout all developmental stages (Imayoshi et al., 2006, 2008). By using this transgenic animal, neuroblast migration along the implanted hydrogel tract was investigated in the brain and the fate of the migrating neuroblasts determined following differentiation. Our approach is summarised in Figure 1.

## Materials and Methods

### Peptide Synthesis

Detailed peptide synthesis was reported in our previous papers (Del Borgo et al., 2013; Kulkarni et al., 2016; Motamed et al., 2016). Briefly, the hydrogel consists of 90% tri-peptide (Ac-β A^*^(C_14_)-β K-β A-OH), where C_14_ alkyl chain was attached to the first amino acid by reducing azide (Motamed et al., 2016), and 10% RGD peptide (Ac-β A^*^(C_14_)-β A^#^ (RGD)-β K-OH (Kulkarni et al., 2016).

### BDNF Release From the Hydrogel

Ten microliters of BDNF full protein (13.5 kDa) stock (R&D Systems) with a concentration of 25 μg mL^−1^ was dissolved in 20 μL phosphate-buffered saline (PBS) to reach a final concentration of ~0.0083 mg mL^−1^. 0.3 mg of the optimized peptide containing 10% RGD peptide and 90% peptide was added to the BDNF solution to reach a final concentration of 10 mg mL^−1^ to form a hydrogel (Hook et al., 2004). The formed hydrogel was then incubated overnight. Three hundred microliters PBS was added on top of the hydrogel and the samples were incubated at 37°C. BDNF release was determined by taking 30 μL aliquots of PBS on top of the hydrogel at different time points and the solution was topped up to keep the volume constant over the course of the assay. Samples were analysed by analytical HPLC (Agilent HP1100), fitted with an Agilent 1100 variable wavelength UV detector. All samples were injected into the HPLC and were run in a system using gradient of solution A (0.1% trifluoroacetic acid (TFA) in water) to solution B (0.1% TFA in acetonitrile), using the method 5% B to 95% B in 20 min. BDNF was monitored by absorbance at 254 nm. All conditions were repeated in triplicate. The amount of released BDNF was quantified by integrating the area under the peak at the retention time of 8.2 min. The released BDNF from the hydrogels at each time point was determined by converting the relevant HPLC peak area to concentration, using a calibration curve (Fon et al., 2014b).

### Nestin-CreER^T2^:R26eYFP Transgenic Mice

In this study Nes-CreERT2 line 5.1: Rosa26-eYFP transgenic mice were used to track the migration of NSCs residing in the SVZ (Imayoshi et al., 2006, 2008; Xing et al., 2014). All animal experiments, approved by the ethics committee of the Florey Institute of Neuroscience (Parkville, VIC, Australia), were performed in accordance with the National Health and Medical Research Council guidelines. To induce recombination, tamoxifen (40 mg mL^−1^ in corn oil) was induced by oral gavage at a dosage of 300 mg/kg. Gavaging was repeated for 4 consecutive days (Xing et al., 2014).

### Hydrogel Preparation

Hydrogels were formed in a sterile environment with UV sterilized peptide powder and sterile PBS and BDNF. Optimized peptide containing 10% RGD-peptide and 90% C14-peptide was dissolved in PBS to reach a concentration of 10 mg mL^−1^. Optimisation was carried out using a cell attachment assay using SN4741 cells (Figure S1). The optimised hydrogel was characterised using and Anton Paar rheometer (Figure S2). For BDNF-loaded hydrogel, BDNF protein (0.0083 mg mL^−1^) was also added to the hydrogel. With reference to mouse atlas (AP 1.1 mm), the hydrogel should be 2.3 mm long to be able to hit the SVZ. Since a 23 g needle with inner diameter of 0.337 mm was used, the required volume of hydrogel considering the density of hydrogel was calculated to be 2.4 μL. To ensure that the formed hydrogel is sufficient to cover the whole area from the SVZ to the cortex a final volume of 3 μL was used. Prior to implantation, 3 μL of hydrogel was loaded into a modified 23-gauge needle. The loaded hydrogels were implanted 5 min after loading to ensure stable hydrogel formation. To ensure that the needle tip did not cause additional injury to the brain, the needle tip was cut and the needle was polished to yield a round and smooth edge.

### Hydrogel Implantation

Implantation of hydrogel was performed 3 days after the final gavaging. Sixteen adult male transgenic mice (average age of 13 weeks) were divided into four mice per experimental condition. They were used to study the change of astrocyte and microglia in response to hydrogel implants/shame injections and also to investigate neuroblast migration along the hydrogel with and without loaded BDNF. Pre-anaesthesia injection was performed intraperitoneally using 0.1 mL atropine (Pfizer) and 0.2 mL xylazine (Troy Laboratories) in 0.7 mL saline (Baxter); 0.001 mL g^−1^ of mouse. Anaesthesia was then induced with the inhalation of 1% isoflurane followed by reducing to 0.5%, which was maintained during the surgery. In order to disrupt the SVZ, the hydrogel implantation was performed at 1.0 mm anterior of bregma, 2.0 mm laterally from the midline of the skull at an injection angle of 25 degrees, with the needle being tilted toward the midline in the coronal plane into the left hemisphere. A needle injury only (sham injection) was created following the same procedure as the hydrogel implantation method. The sham injection served as a control to investigate the cellular response following injury at the same coordinate into the right hemisphere of the animals.

To determine cellular responses, mice were culled 7 days and 35 days after the implantation with 0.1 mL Lethabarb (sodium pentobarbitone) in 0.9 mL saline; 0.006 mL g^−1^ of animal and perfused first with PBS (0.2 M) and then with 4% PFA (paraformaldehyde) in PBS. The brains were removed and fixed in 4% PFA for 2 h and then transferred to a 30% sucrose solution until the brains sank to the bottom of the tube. Brains were then frozen with dry ice and stored at −80°C. The brains were serially sectioned in the coronal plane using a cryostat (Leica) with a thickness of 30 μm (micron) and then the sections were air dried for 1 h prior to storage (Zhou et al., 2016). In total 60 sections were collected on 10 slides per each mouse to cover the whole thickness of implant.

### Immunohistochemistry

Brain sections were fixed with 4% paraformaldehyde (Sigma-Aldrich) for 1 min at room temperature and then rinsed with PBS for 3 × 5 min. The brain sections were then permeabilised in 0.3% Triton-X100 for 5 min and washed in PBS for 3 × 5 min. The non-specific antibody binding was blocked with 10% NGS (Normal goat serum) (Vector Laboratories) including 1% BSA (Bovine serum albumin) (Sigma) and 0.2% Tween20 in PBS for 1 h at room temperature followed by a PBS wash. Brain sections were then stained with several antibodies: rabbit anti-Iba1 (1:250) (Wako Pure Chemical Industries) (microglia marker), rabbit anti-GFAP (1:1000) (Dako) (astrocyte marker), chicken anti-GFP (1:200) (Abcam), rabbit doublecortin (1:400) (Cell Signalling) (DCX; neuroblast and immature neurons marker), mouse anti-NeuN (1:100) (mature neuron marker) (Abcam), and rabbit anti-synapsin 1 (1:100) (Thermofisher) in 1% BSA in PBS at 4°C overnight. The sections were rinsed thoroughly with 0.2% Tween 20 in PBS on the following day (3 × 5 min) and incubated in anti-rabbit Alexa Flour 568 (1:1000), anti-chicken Alexa Flour 488 (1:000) or anti-mouse Alexa Flour 488 (1:000) (Thermo Fisher Scientific) in 0.05% Tween 20 in PBS at 37°C for 1 h. After thorough washing with 0.2% Tween 20 in PBS (2 × 5 min), the sections were counterstained with DAPI (1 μg/ml) (Thermo Fisher Scientific) for 5 min and after additional thorough washing (1 × 5 min) the slides were mounted by coverslip and prepared for fluorescent imaging using a Leica microscope. Images were captured with three fluorescence channels and were merged using ImageJ software (NIH). The boundaries of hydrogel and sham injection were estimated via the accumulation of cells using DAPI staining, due to high levels of brain tissue response to sham injuries and implants. This was revealed by high density of cells (e.g., microglia, astrocytes) with DAPI staining at these boundaries. For cell quantification, the whole length of the hydrogel from SVZ to the top of the brain was taken into consideration. Microglia cells per 10^4^ μm were counted within the hydrogel and compared to the sham injection. The centre of the material tracts was estimated and 100 μm by 100 μm grids were put along the centre line on both sides for quantification (Figures S4e,f). Cells quantification occurred in the same position relative to the needle track in all animals. Astrocyte quantification was performed using the fluorescence intensity from the implant boundary outwards in comparison to a region of the brain away from the implant. The neuroblast migration distance was determined to investigate the ability of the hydrogel to re-direct the neuroblasts from the ventricles. Co-expression of GFP and GFAP was studied by using ImageJ software (NIH). Briefly, a stack image was created by composite GFP and GFAP channel images. A 5 μm wide straight line was drawn across cells of interest. An intensity plot was then generated by the region of interest to study the colocalisation of fluorescence intensity from different channels.

### Statistical Analysis

Statistical analysis was performed on 9 sections for each cell type quantifications. Cell quantifications were expressed as mean ± standard deviation. Equal variances in different groups were confirmed by Levene's Median Test. The groups were then compared using one-way ANOVA with Tukey's *post hoc* testing (GraphPad Prism Version 6.01). *P* < 0.05 was used to determine statistical significance.

## Results and Discussion

### β-Peptide Hydrogel Is Biocompatible in the Brain

**Figure 2:**
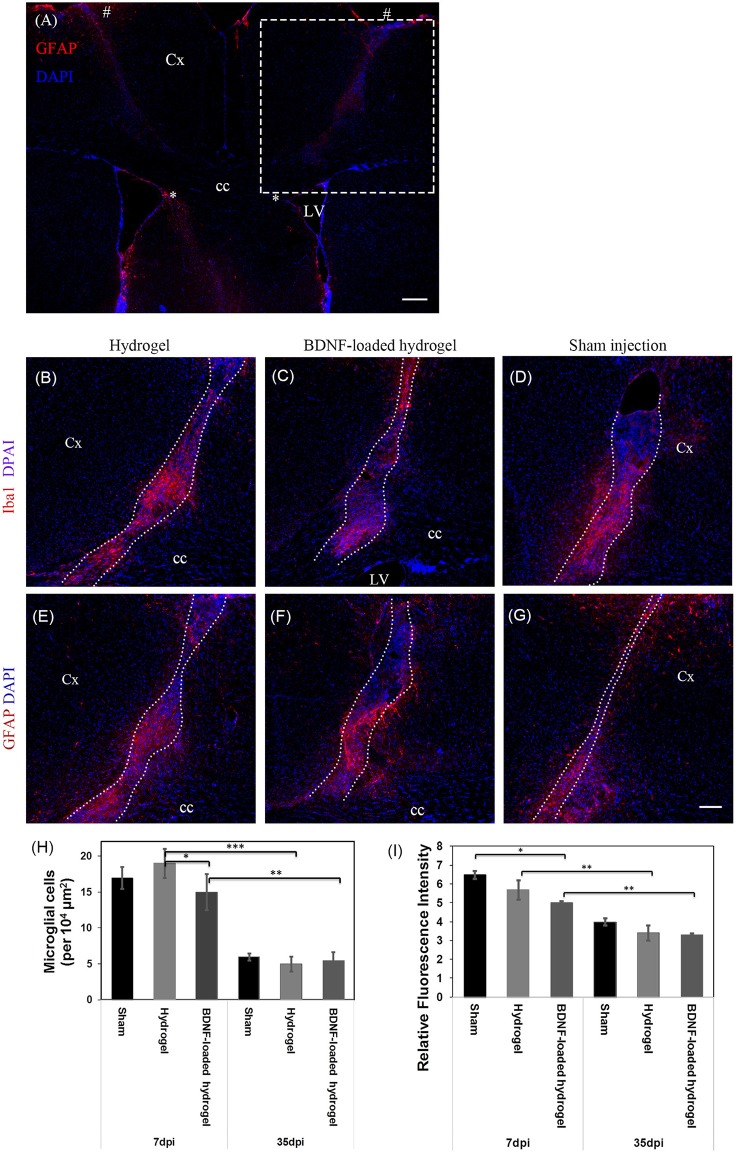
Astrocyte and microglia response to implants and sham injections. **(A)** Low magnification image showing the full track of sham injection (left hemisphere) and hydrogel implantation (right hemisphere). * and # indicate the start and end point of the tract. The dash box indicates the imaging region for **(B–G)**. Dash box not shown in the symmetric left hemisphere. Iba1 positive microglial cells at 7 d, **(B)** Hydrogel; **(C)** BDNF-loaded hydrogel; **(D)** Sham injection; The sections were counter stained with DAPI (blue) to stain all cell nuclei. Astrocyte cells at 7 d, **(E)** hydrogel; **(F)** BDNF-loaded hydrogel; **(G)** sham injection; Cells were stained with GFAP (red) as astrocyte marker and DAPI (blue) for all cell nuclei. **(H)** Number of microglia per 10^4^ μm^2^ around the center of the injection site and infiltrated into the hydrogels. **(D,G)** have been rotated horizontally for comparison purposes. **(I)** Relative GFAP intensity to the uninjured part of the brain (*P ≤ 0.05, **P ≤ 0.01, and ***P ≤ 0.001). Error bars represent standard deviation. Cx, cortex; cc, corpus callosum; LV, lateral ventrical. Scale bar = 200 μm for **(A)** and 100 μm for **(B–G)**. Dotted line indicates the boundary of hydrogel/sham injection tracks. *n* = 4 animals.

The change of astrocyte and microglia level in response to the β-peptide hydrogel was assessed by quantifying the astrocyte and microglial responses. A 23 g stainless steel hypodermic needle was injected into the brain as a control to determine the cellular response to injury caused by the injection of the material with the same sized needle. The change of astrocyte and microglia level was examined at 7 d which corresponds to the time period of peak acute activation (Nisbet et al., 2009) and also at 35 d. Sham injection (needle injury) is commonly used as one of the methods to study brain injury (Bjugstad et al., 2010; Nagamoto-Combs et al., 2010; Kishimoto et al., 2012; Rasouli et al., 2012; Xia et al., 2015). Figure 2A shows the location of the full tract of the sham injection (left hemisphere) and hydrogel implantation (right hemisphere). Figures 2B–D are representative images of the cellular response to injury for β-peptide and the sham injection at 7 d. Microglia cells were observed within 150 μm of the boundary. The number of microglia around the injection and inside the β-peptide hydrogel was counted and the average number of cells per 10^4^ μm presented in Figure 2H. There was a significant increase in microglia at the site of injury at 7 d, where the microglia cell number was almost similar for both the hydrogel and sham injection. Microglial response around the BDNF-loaded hydrogel was significantly lower than the response around the hydrogel and sham injection, which can be attributed to the anti-inflammatory properties of BDNF (Joosten and Houweling, 2004; Fon et al., 2014b), reducing the number of microglia. The number of microglia cells decreased dramatically from 7 to 35 d, most of which were accumulated inside the hydrogel (Figures S4a,b).

The number of astrocytes was determined by relative glial fibrillary acidic protein (GFAP) fluorescence intensity to the uninjured parts of the brain (Figures 2E–G). Astrogliosis was seen within 100 μm of the boundary of sham injection (injury)/implant at both 7 and 35 days which slightly decreased as a function of Htime and distance from the lesion (Figure 2I, Figures S4c,d). Astrocyte cell number within 100 μm of the centre of the lesion was similar for both sham injection and hydrogel. Astrocytes were present along the hydrogel tract, which increased as a function of time, similar to a previous study (Fon et al., 2014a), and there was no evidence of glial scar formation. From observation of the section, the incorporation of BDNF in the hydrogel appeared to reduce the numbers of actrocytes, with the number of astrocytes decreased as a function of distance from the centre of the lesion. An *in vitro* BDNF release study (Figure S3) showed that all the added BDNF was released by 5 days, however we expect this to be more rapid *in vivo* due to the higher surface area of the injected hydrogel tract. There was an increased number of astrocytes toward the centre the BDNF-loaded hydrogel tract which could ultimately play key roles for the survival and migration of neuroblasts (Theodosis et al., 2008).

Overall, the number of microglia and astrocyte found in this study suggest that the hydrogel is biocompatible and integrates well with the parenchyma. While the exogenous BDNF released from the hydrogel is expected to be rapidly released *in vivo*, it suppressed the tissue response and subsequently improving tissue-scaffold integration.

### Neuroblasts Migrate Through the Entire Length of Implanted β-Peptide Hydrogel

**Figure 3:**
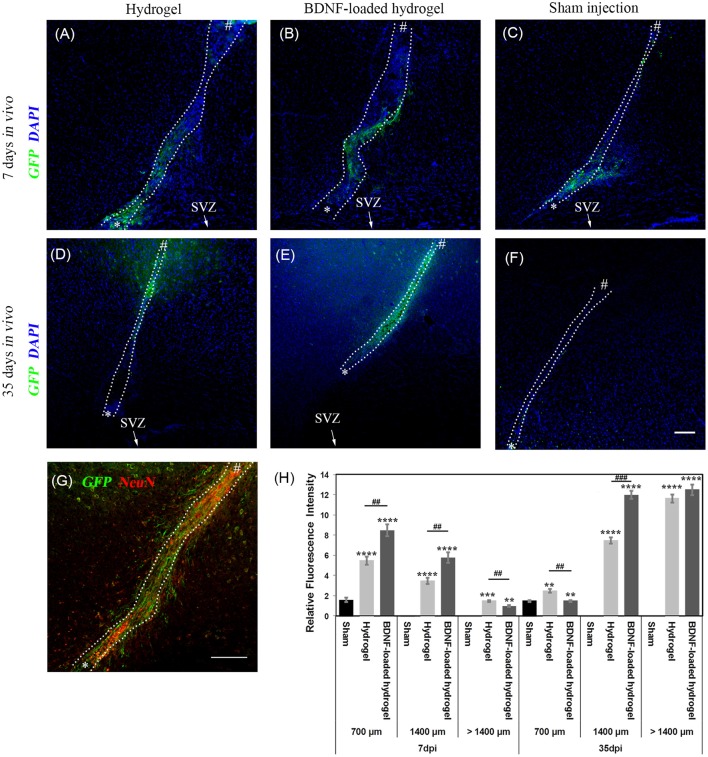
Neuroblast migration within the cortex in response to injury. **(A)** hydrogel at 7 d; **(B)** BDNF-loaded hydrogel at 7 d; **(C)** sham injection at 7 d; **(D)** hydrogel at 35 d; **(E)** BDNF-loaded hydrogel at 35 d; **(F)** sham injection at 35 d; **(G)** high magnification image of neuroblasts at 35 d for the BDNF-loaded hydrogel. GFP positive (green) and DAPI stained nuclei (blue) are shown. Scale bar = 100 μm for A-F. “*” and “#” indicate the start and end points of hydrogel/sham injection tracks, respectively in **(A–G)**. **(H)** Relative GFP fluorescence intensity to the uninjured part of the brain for different distances from the SVZ. *indicate significant difference between sham and hydrogel with or without loaded BDNF groups. # indicate significant difference between hydrogel and BDNF-loaded hydrogel groups. *P ≤ 0.05, ** and ^*##*^*P* ≤ 0.01, *** and ^*###*^*P* ≤ 0.001, ****P ≤ 0.0001. Error bars represent standard deviation. *n* = 4 animals. **(C,F)** were horizontally rotated for easy comparison. Dotted line indicates the boundary of hydrogel/sham injection tracks.

NSCs that originate from the SVZ in Nes-CreER^T2^:R26eYFP transgenic mice are permanently labelled as GFP+ve cells, regardless of the different developmental stages. Therefore, it is possible to identify the migrating cells and their progeny (Imayoshi et al., 2006; Xing et al., 2014; Kulkarni et al., 2016). GFP+ve cell migration was observed in response to injury at 7 d for all studied conditions, where the number of migrating cells and the distance of migration were significantly lower (*P* ≤ 0.01) for the sham injection (Figure 3). GFP+ve cells migrated for a short distance from the SVZ around the lesion due to the injury caused by the sham injection (Figure 3C). In contrast, GFP+ve cells migrated away from the SVZ through the implanted β-peptide hydrogels and were quantified in terms of relative fluorescence intensity as a function of distance from the SVZ at 7 days and 35 days (Figure 3H).

GFP+ve cells were observed along the hydrogel tract. At 7 days, the number of migrating cells along both the hydrogel tracts decreased as a function of distance from the SVZ and the migration was confined along the hydrogel (Figures 3A,B). At 35 days, the migration was uniform (Figure 3E) with greater numbers of cells for the BDNF-loaded hydrogels (Figure 3G). This is reminiscent of neuroblast migration through the RMS (Ghashghaei et al., 2007). After 35 days, GFP+ve cells reached the end of the hydrogel tract at the cortex and migrated to the surrounding tissue, forming clusters. Figure 3E shows that the migration along the BDNF-loaded hydrogel was more abundant and cells migrated in greater numbers.

### Neuroblasts Differentiate Into Neurons and Astrocytes

NSCs differentiate into various types of cells through their developmental stages. NSCs residing in the SVZ initially express GFAP. They then differentiate into migrating neuroblasts and can be detected as immature DCX+ve neuroblasts. At the end of the migration, they either differentiate into cells expressing GFAP, oligodendrocytes or mature into neurons, expressing the mature neuron marker, NeuN (Ming and Song, 2011; Faiz et al., 2015). In order to understand the stage of maturation of the GFP+ve cells along the β-peptide hydrogel, brain sections were stained with different markers at 7 and 35 d.

**Figure 4:**
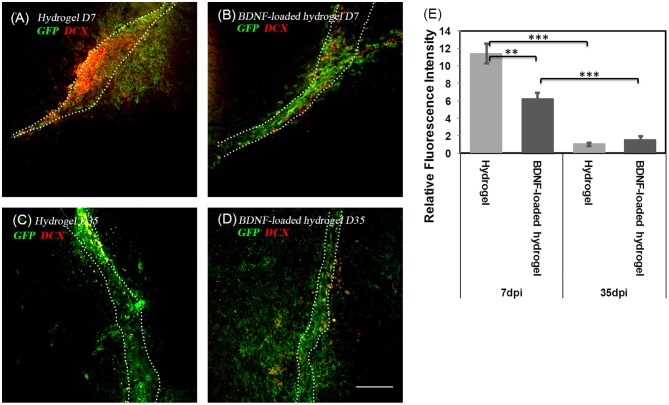
The co-localization of GFP+ve cells and immature neuroblasts at two time points; **(A)** hydrogel at 7 d; **(B)** BDNF-loaded hydrogel at 7 d; **(C)** hydrogel at 35 d; **(D)** BDNF-loaded hydrogel at 35 d. Cells were stained with GFP (green) and DCX (red). Scale bar = 100 μm for all images. **(E)** Relative DCX intensity to the uninjured part of the brain (**P ≤ 0.01 and ***P ≤ 0.001). Error bars represent standard deviation. *n* = 4 animals. Dotted line indicates the boundary of hydrogel/sham injection tracks.

To investigate the fate of NSCs, DCX staining, a marker for migrating and immature neurons was performed. At 7 d, the majority of migrating cells along the hydrogel expressed DCX, showing that the GFP+ve cells are in their immature migrating state (Figures 4A,B). However, after 35 d, the population of DCX+ve cells significantly decreased (Figures 4C–E).

**Figure 5:**
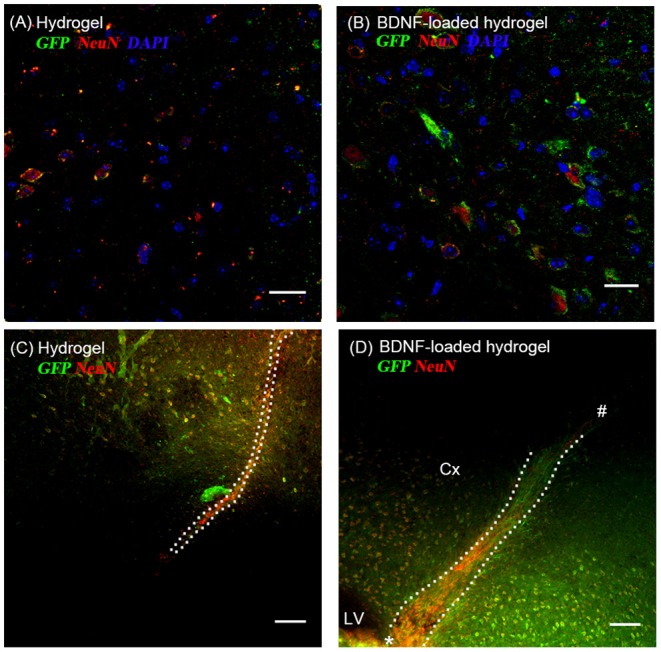
The co-localization of GFP+ve cells originated from the SVZ and mature neurons at 35 d; **(A,C)** hydrogel; **(B,D)** BDNF-loaded hydrogel. Cells were stained with GFP (green), NeuN (red), and DAPI (blue). **(D)** “*” and “#” indicate the start and end point of hydrogel track, respectively. Scale bar for **(A,B)** = 25 μm. Scale bar for **(C,D)** = 100 μm. Dotted line indicates the boundary of hydrogel/sham injection tracks. Cx, cortex; LV, lateral ventrical. *n* = 4 animals.

Most GFP+ve cells are co-localized with NeuN+ve cells (Figures 5A,B), indicating that the neuroblasts differentiated into neurons by 35 d. This co-localization is more abundant at the end of the hydrogel tract than the start (Figures 5C,D), where most of the GFP+ve cells are not stained with NeuN+ve, showing that they are most likely immature neurons or differentiated into astrocytes or oligodendrocytes. The migration stream was narrow for the hydrogel and most of the cells migrated toward the surrounding tissue at the end of their migration. From observation, large numbers of migrating cells remained along the BDNF-loaded hydrogel, suggesting that this matrix was more permissive for substantial neuroblast migration. Significantly, most of the newly generated neurons were Syn1 positive suggesting the formation of synapses (Figure S5).

While a number of previous studies have utilised matrices to promote neuroblast migration, the length of migration has been limited to the first quarter of the implant length (Fon et al., 2014a; Clark et al., 2016). Our previous work using injectable gelatin hydrogels was degraded quickly and was cleared after 3 weeks, and therefore was unable to promote neuroblast migration over longer periods. The number of neuroblasts around the gelatin matrix decreased from 7 to 21 d, while at the same time the number of neurons increased, which may be due to differentiation of migrated neuroblasts to neurons (Fon et al., 2014a). However, there was no conclusive evidence of this, because of the inability to conclusively map neuroblast progeny.

### Migrating Neuroblasts Are Co-localised With Astrocytes

Astrocytes play a pivotal role in neuroblast migration through the RMS (Gengatharan et al., 2016). At the early postnatal stages, they are located at the border of the RMS, secreting VEGF to induce vasculature, which is required for neuroblast direction toward the olfactory bulb (Ma et al., 2009). Later on, their branches are elongated along blood vessels and in close proximity to migrating neuroblasts (Bovetti et al., 2007; Whitman et al., 2009), enveloping the migrating cells and blood vessels and forming a glial tube to isolate the neuroblast migration from the surrounding tissue (Snapyan et al., 2009). They also release EGF, soluble melanoma inhibitory activity (MIA) protein and glutamate, which are crucial for neuroblasts to exit from the SVZ, neuroblast migration and survival of neuroblasts (Mason et al., 2001; Caldwell et al., 2004; Platel et al., 2010). In addition, astrocytes trap BDNF through high affinity tropomyosin receptor kinase B receptors, inducing the neuroblast stationary phase, thus regulating the migration process (Snapyan et al., 2009). Previous studies have shown astrocytes distribute at the scaffold boundary and use the scaffold orientation to assist the guidance of neurite extension (Deumens et al., 2004; Schnell et al., 2007; Yucel et al., 2010).

**Figure 6:**
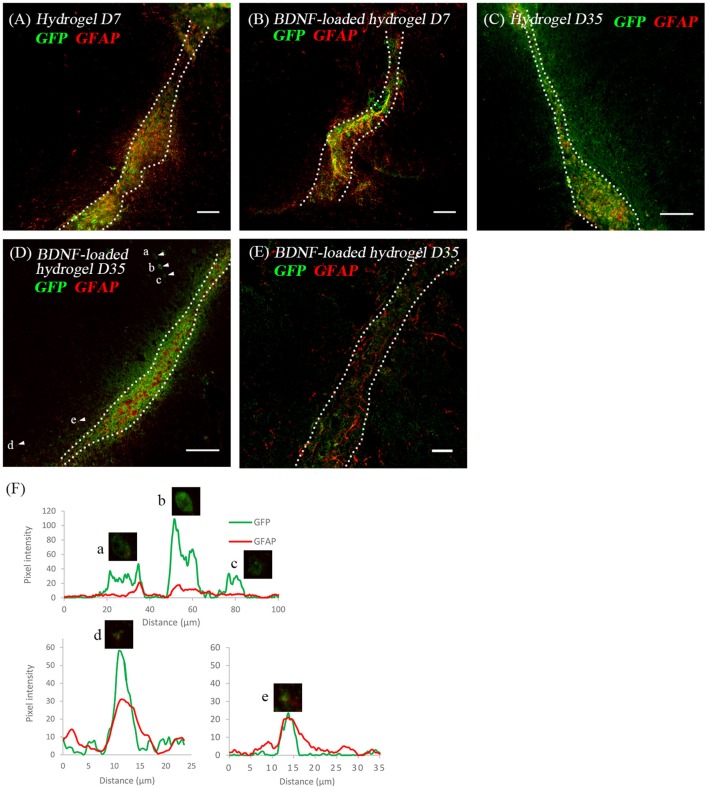
The co-location of GFP+ve cells and astrocytes at two time points; **(A)** hydrogel at 7 d; **(B)** BDNF-loaded hydrogel at 7 d; **(C)** hydrogel at 35 d; **(D)** BDNF-loaded hydrogel at 35 d; **(E)** high magnification image showing the co-existence of GFP+ve cells with astrocytes. Scale bar = 20 μm. Cell were stained with GFP (green) and GFAP (red). Scale bar = 100 μm for **(A–D)**. **(F)** Fluorescence intensity map shows examples of cells that expressed GFP only (Cells a, b, and c) and those that co-expressed GFP and GFAP (Cells d and e). Intensity is presented as a function of moving through the cells (distance). Dotted line indicates the boundary of hydrogel/sham injection tracks. *n* = 4 animals.

Considering the critical role of astrocytes in the RMS, we investigated the astrocyte-neuroblast co-location along the implanted β-peptide hydrogel tract. Figures 6A,B shows the presence of astrocytes and GFP+ve cells along the hydrogel tract at 7 days. After 35 days some of the neuroblasts differentiated into astrocytes (Figures 6C,D). There is a mixture of GFP+ve cells co-localised with GFAP+ve cells (Figures 6D,F) and some GFP+ve cells in close proximity to the GFAP+ve cell processes (Figure 6E). After 35 days, a mixture of astrocytes including both GFP+ve astrocytes and local astrocytes were present along the hydrogel tract (Figures 6C,D). Astrocytes formed a pathway along the direction of the BDNF-loaded hydrogel tract, leading to a greater number of migratory cells (Figure 3G). At day 35, it was also evident that endogenous BDNF was localised along the hydrogel tract which would also act to facilitate migration of the neuroblasts (Figure S6).

### Hydrogel Persisted in the Brain at 35 Days

**Figure 7:**
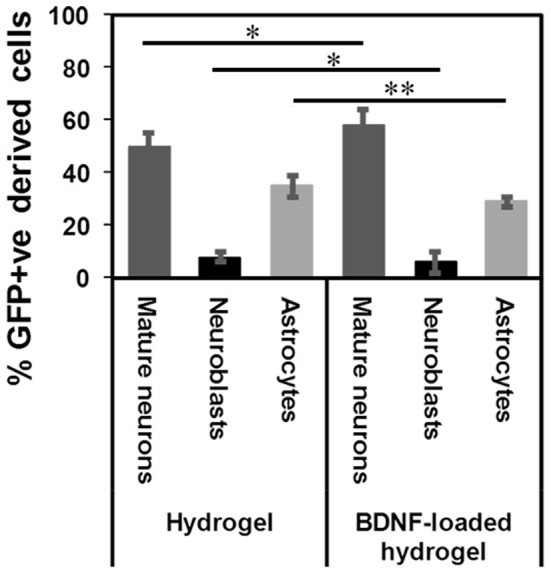
GFP+ve cells differentiated into different cells at 35 d: mature neurons (dark grey), neuroblasts (black), and astrocytes (light grey) (*P ≤ 0.05, **P ≤ 0.01). *n* = 4 animals.

β-peptides are proteolytically stable providing long-term support for cell migration and differentiation (Aguilar et al., 2007; Kulkarni et al., 2016; Motamed et al., 2016). In contrast, more commonly used self-assembling α-peptide hydrogels are degraded by 14 d (Fon et al., 2014a; Li et al., 2014). Figure S7 shows that the β-peptide hydrogel remained intact in the brain. The tract contained numerous types of cells at 35 d. 50 ± 5% of GFP+ve cells differentiated into mature neurons along the hydrogel tract, while 8 ± 2% of cells still had a migrating DCX+ phenotype and 35 ± 4% had differentiated into astrocytes (Figure 7). Although the proportion of differentiation was independent of the presence of BDNF, the number of migrating cells and consequently the number of mature neurons were significantly higher for the BDNF-loaded hydrogel. This may be attributed to the initial effect of released BDNF in suppressing the tissue response.

## Conclusions

Utilising *NestinCreER*^*T*2^*:R26eYFP* transgenic mice for the indelible labelling of neuroblasts originating in the SVZ, the impact of a long-lasting β-peptide hydrogel on the migration of neuroblasts and their progenies in a healthy brain was determined. After 7 days, the number of migrating neuroblasts along the hydrogel tract decreased as a function of distance from the SVZ and the migration was confined along the hydrogel tract. The addition of exogenous BDNF did not affect the number of migrating neuroblasts along the hydrogel tract. However, exogenous BDNF attenuated the tissue response and the neuroblasts migrated in a more uniform pattern. After 35 days neuroblasts migrated along the hydrogel tract for some distance and then left the hydrogel tract. However, in the BDNF-loaded hydrogel, the neuroblasts tended to remain along the hydrogel tract. Fate mapping showed that the neuroblasts differentiated into Syn1-positive neurons and astrocytes.

## Data Availability Statement

The raw data supporting the conclusions of this manuscript will be made available by the authors, without undue reservation, to any qualified researcher.

## Ethics Statement

All animal experiments, approved by the ethics committee of the Florey Institute of Neuroscience (Parkville, VIC, Australia), were performed in accordance with the National Health and Medical Research Council guidelines.

## Author Contributions

JF, DF, M-IA, MD, PC, and TM conceptualised the ideas. SM, MD, KK, and KZ performed the experiments. SM wrote the first draft of the manuscript. All authors provided revisions, designed the experiments, and analysed the data.

### Conflict of Interest

The authors declare that the research was conducted in the absence of any commercial or financial relationships that could be construed as a potential conflict of interest.
