# Supplementary material for: Migration and Differentiation of Neural Stem Cells Diverted From the Subventricular Zone by an Injectable Self-Assembling β-Peptide Hydrogel
Source: Front Bioeng Biotechnol. 2019 Nov 8;7:315. doi: 10.3389/fbioe.2019.00315 (PMC6856563; doi:10.3389/fbioe.2019.00315)
Supplement: Supplementary file 1 [file Data_Sheet_1.pdf]

Supplementary Material

1 *in vitro* characterization and optimisation of hydrogel

To determine the optimised scaffold for high cell attachment a set of composite hydrogels with different percentages of RGD-peptide and C_{14}-peptide were prepared and assessed for dopaminergic neuronal progenitor cells (SN4741) attachment. Cells viability and morphology of the attached cells were investigated by fluorescence imaging of cells stained with calcein AM / ethidium after 2 days. Cell attachment with elongated morphology was enhanced by increasing RGD-peptide concentration up to 10% and decreased by further increases in the RGD-peptide concentration, which was attributed to epitope saturation. Therefore, hydrogel containing 10% RGD-peptide was used for further investigation.

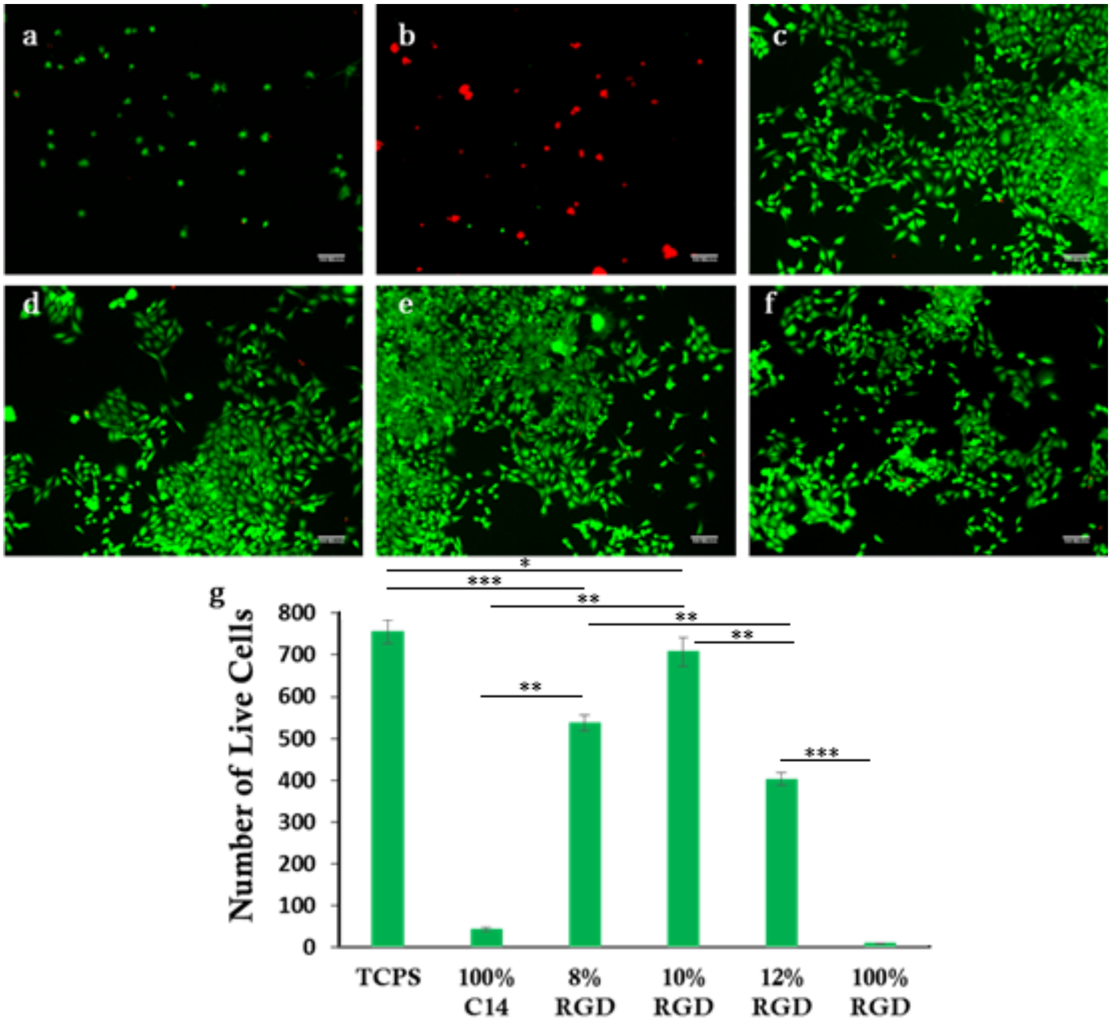

Figure S1. SN4741 cells stained with calcein AM and ethidium bromide after 2 days on (a) C_{14}-peptide hydrogel; (b) RGD-peptide hydrogel; (c) TCPS; (d) hydrogel containing 8% RGD-peptide and 92% C_{14}-peptide; (e) hydrogel containing 10% RGD-peptide and 90% C_{14}-peptide; (f) hydrogel

containing 12% RGD-peptide and 88% C_{14}-peptide. Live cells were stained with calcein AM as green cells. Dead cells were stained with ethidium homodimer as red round cells. Scale bar 100 µm. (g) Cell viability on hydrogels with different concentrations of RGD-peptide and C_{14}-peptide after 2 days culture (* $P \leq 0.05$, ** $P \leq 0.01$, ***$P \leq 0.001$)

2 Physical characterization of the optimised scaffold

Rheological studies were conducted using an Anton Paar rheometer (Physica MCR 501) with an 8 mm parallel plate. The temperature was maintained at 35 °C for all experiments. Peptide samples were dissolved in PBS buffer to give a final concentration of 10 mg ml-1. Experiments were immediately performed with a plate gap of 0.1 mm. The gelation of the peptide over time was examined for 2 hours and the storage (elastic) and loss (viscous) moduli were reported as a function of time. When the hydrogel reached an equilibrium state, its response to frequency variation was examined. All experiments were repeated three times and the average values were reported.

The stiffness of the optimised scaffold containing 10% RGD peptide was characterised using rheology. The optimised hydrogel has a storage modulus of about 1.4 kPa, in an acceptable range similar to brain tissue. The hydrogel is stable over a wide range of frequencies (0.1-100 rad s-1).

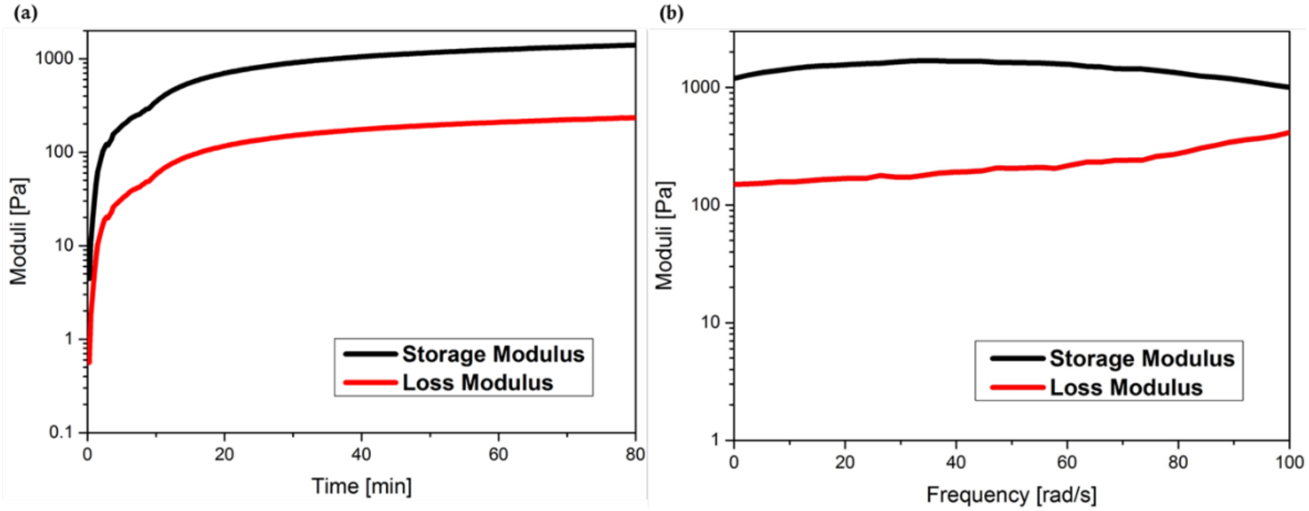

Figure S2. Rheological behaviour of the optimized hydrogel containing 10% RGD-peptide at 10 mg mL^{-1} concentration; (a) evolution of hydrogel formation at constant 1% strain and 1 Hz frequency; (b) Frequency sweep at a range of 0.1-100 rad s-1 at constant 1% strain.

3 BDNF release from the hydrogel

To utilize the maximum capacity of BDNF, encapsulation of the protein in the hydrogel is preferred in comparison to direct injection, since the hydrogel prolongs its persistence by releasing it over time. The BDNF release from the hydrogel is diffusion-driven with an initial burst release of about half of the encapsulated amount within the first day, which can be attributed to the hydrated nature and high porosity of the hydrogel. The total amount of BDNF was released from the hydrogel after 5 days. This release profile only demonstrates protein release from hydrogel over time and is an approximation of the release kinetics in vivo. The implanted scaffold has a higher surface area and protein can be released faster through the outer layers of scaffold. Although the scaffold was

implanted as a pre-formed hydrogel, it is postulated that the protein release would be higher due to the high shear rate from surrounding tissue while implanting.

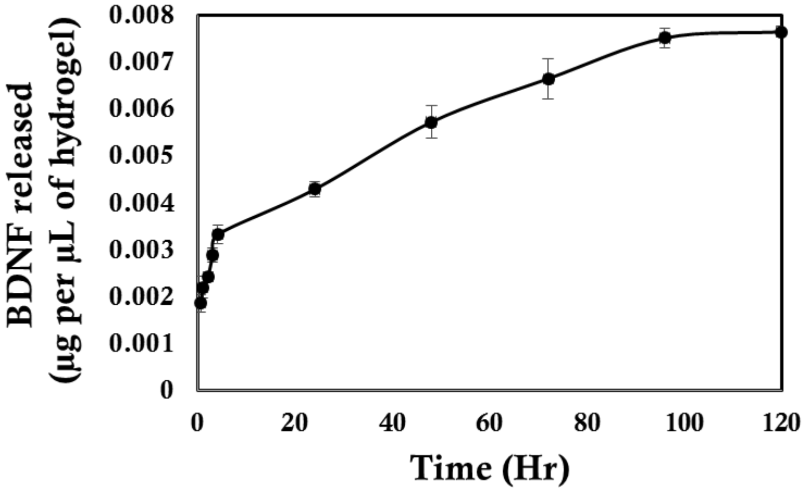

Figure S3. The amount of BDNF released in vitro (μg per μL of hydrogel) from the optimised hydrogel at different time points.

4 Hydrogel Biocompatibility – Microglia and Astrocytes Response

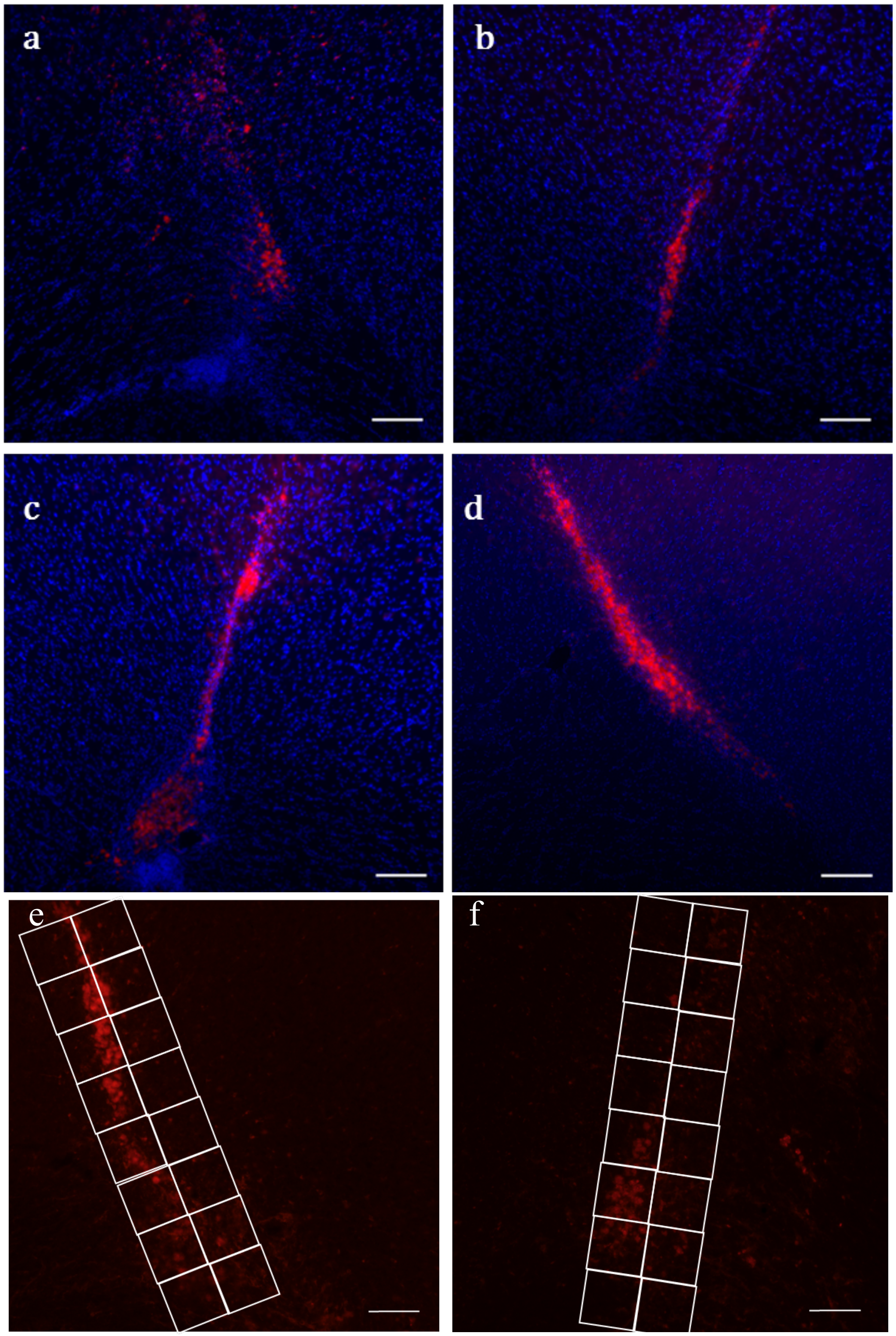

Figure S4. Inflammatory Response. Microglial activation at 35 d, (a) Hydrogel; (b) BDNF-loaded hydrogel; Cells were stained with Iba1 (red) as microglia marker and DAPI (blue) for all cell nuclei. Astrocyte activation at 35 d, (c) hydrogel; (d) BDNF-loaded hydrogel; Cells were stained with glial GFAP (red) as astrocyte marker and DAPI (blue) for all cell nuclei. Quantification of microglia at 7d (e) hydrogel; (f) BDNF-loaded hydrogel. Cells were stained with Iba1 (red) as microglia marker. Squares = 100 μm x 100 μm. Scale bar = 100 μm.

5 Synapsin staining

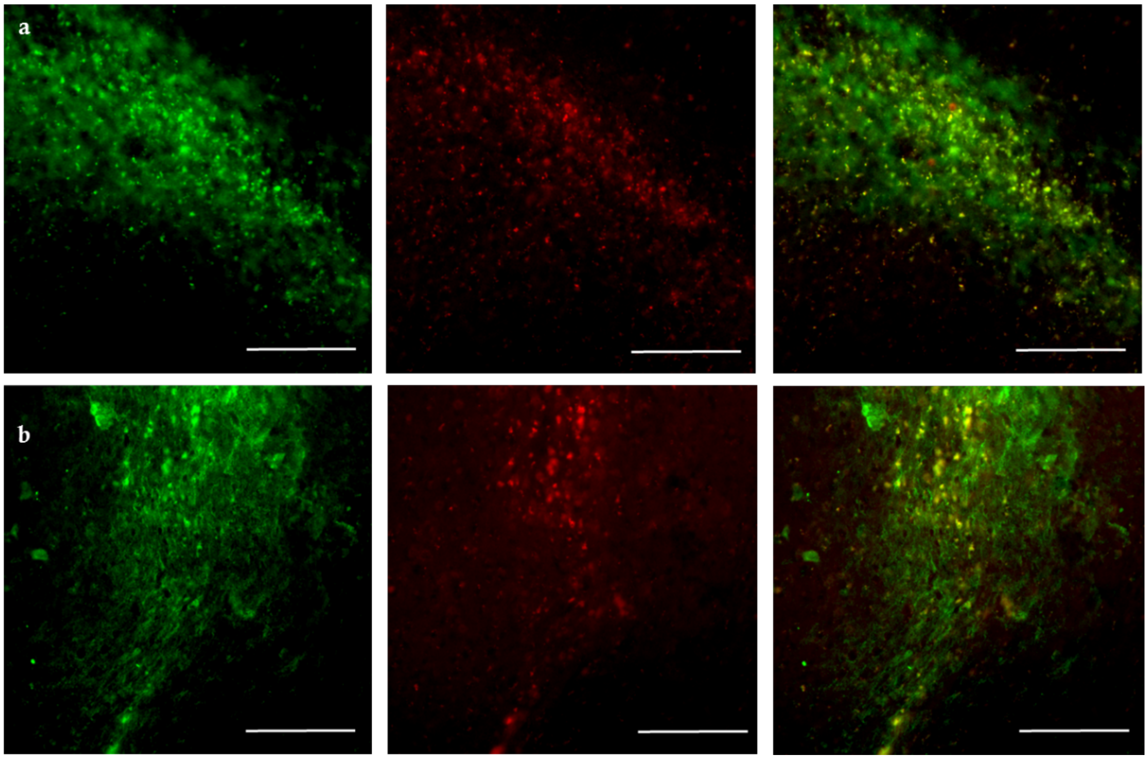

Figure S5. GFP+ve cells at 35 d showing synapsin positive staining; (a) hydrogel; (b) BDNF-loaded hydrogel. Cell were stained with GFP (green) and synapsin antibody (red). Scale bar = 50 µm

6 Existence of endogenous BDNF

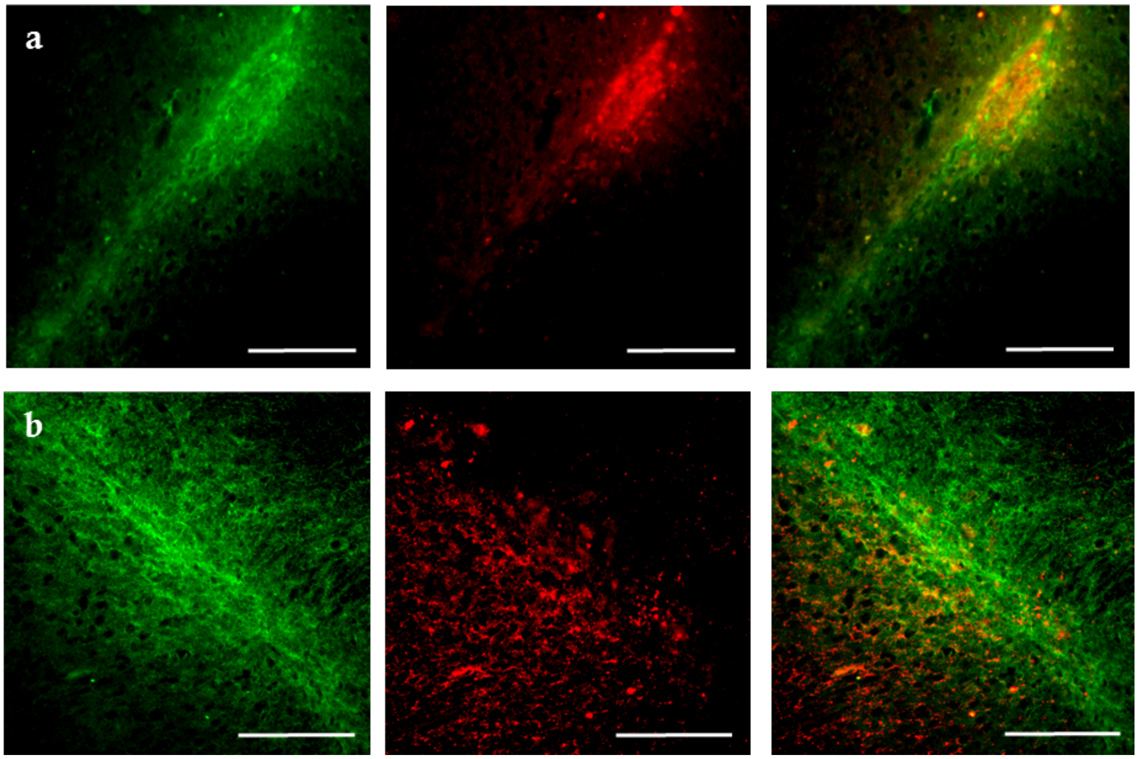

Figure S6. The co-existence of GFP+ve cells and BDNF at 35 dpi; (a) hydrogel; (b) BDNF-loaded hydrogel. Cell were stained with GFP (green) and anti-BDNF (red). Scale bar = 50 μm

7 Hydrogel persistence in the brain

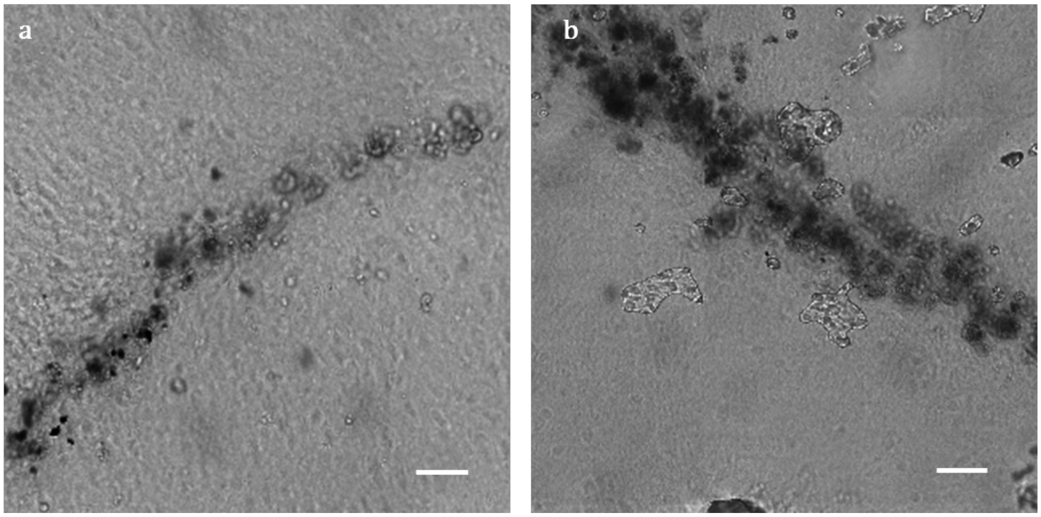

Figure S7. Hydrogel persisted in the brain at 35 d. (a, b) Bright field images of hydrogel in the brain at 35d; scale bar = 20 μm.
